# Extra-Limites

**Published:** 1841-10-01

**Authors:** 


					1841] ( 593 )
EXTEA-LIMITES,
CHUSAN.
[Communicated by Sir W. Burnett.]
Extract from the Nosological Return of H. M. Snip Volage,
BETWEEN THE 1ST JuLY 1S40, AND THE 30th SEPTEMBER 1840.
On the 28th July, we arrived at the new British possession, on the coast of
China, "Chusan"?an island situated in the 30th degree of North latitude, and
about 9 or 10 miles from the nearest part of the main land of China. It had
been in the possession of the British since the 6tli of the same month.
The capital town of the island is named "Ting-Hae," about an English mile
from the sea-side. There is a small suburb close to the shore, between which
and "Ting-Hae," country intervenes, flat, intersected by canals, and overgrown
with rice and millet seed. Ting-Hae is also built on a portion of this flat?and
the flat extends from it, in every direction, towards the country three or four
miles; except two isolated spots, where the ground is naturally raised, the whole
of this flat is so far below the level of the sea, that even the roofs of the houses
are not to be seen from the deck of a frigate. The part where the suburb ig
built is somewhat higher, and forms an embankment against the sea, formed, I
have no doubt, by the hand of man, but cemented into the appearance of nature
by long lapse of time, and the numerous embankments pervading many parts of
the neighbouring islands bear me out in the statement?so that I am satisfied
the sites of Ting-Hae and the adjoining country have been, in some remote pe-
riod, cut off from the sea.
In the month of July the country was in the height of cultivation in its vari-
ous stages?no barren spot to be seen. At a short distance, the view of the
country was like a vast collection of garden-grounds.
Much of the soil of the flat around the town was artificial, and in its then
moist state had been evidently lately flooded by a most perfect system of irriga-
tion, so that a stick penetrated this soft and rich soil to the extent of 14 or 15
'nches.
Ting-Hae itself is a most wretched town, occupying a circuit over three miles,
surrounded by a wall built of stone and brick about a foot and a half thick, in-
side of which is a mound of earth, supported in its outer circuit by the wall.
On the inner side, this mound forms a slope, with a plain on the top. The wall
then ascends forming a parapet, breast high, in which are numerous embrasures
for matchlocks and archery, and a few longer ones near the gates for guns?a
few also of which, old and* honeycombed, were fixed there, secured on wooden
stands, such as are used in England to rest porter casks on. The height of the
^all in its outer circumference varies from 25 to 15 feet, in many parts dilapi-
dated. The houses are low, none higher than the wall. Attached to each
house is placed, without regard to decency, a necessary-house?in some parts
two or three to each, all open and exposed. The streets are narrow, paved on
e|ther side, and in the centre is a gutter covered by a flag-stone, ill-concealing
either the smell or view of subjacent filth, now disgusting to look at, and per-
fectly nauseating by its stench. Every house was also provided with one or
^ore immense earthen jars, also receptacles for filth and excrement in its more
fluid form. All these things, taken in connexion with the surrounding soil,
No. LXX. Q q
r\
V \
594 Extra-Limites. [Oct. 1
prove that manure of every description was carefully preserved, the ingredients
for which were, though then in an incipient stage, fearfully, as far as health was
concerned, abounding and rapidly decomposing. Exuberant vegetation was al-
ready shooting from the muddy banks of the canals; and, even in the midst of
the stagnant waters of which, was also to be seen some show of it. All this of
course, unless provided against, was rapidly to end in rottenness. The thermo-
meter was varying from 84? to 94?, and the atmosphere was, in the more con-
fined and less ventilated parts, already sensibly loaded with deleterious effluvia,
which the shockingly pent-up position and arrangement of the town rendered
it difficult to dissipate.
In whatever part of the town one turned, the decomposition and consequent
stench were offensive. If, in three weeks of July, such was the state of things,
what must August and September produce ? Without some great change, surely
epidemic and contagious disease; and such has been the fact. That the state
then existing should be changed, there can be no doubt, though the mode of
doing it admits of some. At least before leaving "Ting-Hae" I saw people at-
tempting to cleanse the town in a manner which I should not have done. I saw
them clearing some of these sinks of filth, with the water still in them, which I
think must tend to further putrefaction.
The sickness (intermittent fever and dysentery) among the troops has been
very great, and mortality to a fearful extent is now among them. Fever and
dysentery have also pervaded the fleet, which certainly cannot be attributed to
the causes above enumerated.
I do not consider that extensive sickness is ever, or hardly ever, to be attribu-
ted to one sole, isolated, cause, and I believe that the sickness now at Chusan is
not to be attributed to those causes above (bad as they are) alluded to.
That low swampy grounds, such as these, produce intermittent fever, one need
not come to Chusan to be assured of; but we have frequent assurances that ex-
halations from putrid and corrupting matter may be dwelt among without ende-
mic and widely-spreading fatal dysentery. The same disease pervading those
both on shore and on ship-board, may be attributed to the same causes, many
of which were common to both services. The water, for instance, with which
?we were both supplied for the first weeks after the occupation of Chusan, was
decidedly bad: it was procured among the swampy ground, smelt badly, and had
a rotten putrescent taste.
The evaporation from the decomposing manured rice-grounds, spread through
some considerable space of the atmosphere, so as to affect numbers within its
influence more or less.
The climate was new to all?so far the causes were common to both services.
Those on shore were of course, from their proximity, more liable to feel the ef-
fects in a far more intense degree than those on ship-board, and more remote;
and besides these causes, to those on shore, were to be superadded to others-
The supply of fresh provisions to the troops was scanty and bad, and even the
salt meats, both beef and pork, were so bad as to be considered unfit for use.
Again, on taking possession of Chusan, one of the most abounding things
there, was "sam-shee," an ardent spirit procured by fermentation from rice and
millet-seeds, more generally the latter. This is a most deleterious spirit; its
effect in producing drunkenness is most rapid, and the drunkenness produced by
it, is attended in the first degree with furious madness, and afterwards with cor-
responding depression, or rather horror, and almost invariably leaving a loW
fever of some days' duration after it. This spirit, I much fear, was used by the
troops to a very unmeasured extent. The sailors had not the same opportuni-
ties.
Another cause acting on those on shore, which we had not, was independent
of the noxious effluvia, the confined and ill-ventilated situation of the town.
Their duties were not, I should say, so heavy as those on ship-board.
1841]
Ckusan. 595
I have gone thus far out of the direct path of the immediate state of the health
of the ship's company, as I think it is probable the general state of health of the
force ma)' become a matter of public importance in England, and my having
been on the spot, and knowing personally of all the circumstances I have nar-
rated, may render such detail, Sir, agreeable to you. The climate of the Chusan
Islands, I should say (remove the causes given above), is fine and healthy. The
Volage left the Chusan Islands on the 1st August to proceed northward on the
Coast of China to the gulf of Pe-chili-li. The highest latitude we attained, was
the 40th degree. Along the coast we had the weather generally fine, and tem-
perate, the thermometer varying during the period (August and September) from
60? to 78??these being the extremes?the average 72?. The winds along the
coast hung steadily to the southward and westward and the entrance of the gulf
?this being the direction of the regular monsoon: and though the monsoon is
said not to extend beyond the 28th degree of latitude, yet the period of July or
August has been always selected to make the passage up to the Gulf of Pe-chili-
li, and in the few trials that have been made, the southerly winds have invariably
blown home to the entrance, while, on the other hand, the passage down again
to the South, has been always made with a favorable or North wind between
the close of September and April, that is, during the period of the northerly
monsoon. We returned at the close of September and found it so.
The squadron, which proceeded to the gulf, consisted of the Wellesley, with
the flag of Admiral Elliot, the Blonde, Volage, Modeste, Pylades, a steamer and
two transports. In all these ships, except ourselves, abdominal disease prevailed
extensively?in some instances assuming the dysenteric form, and fatal.
I saw one of these cases, the second Lieutenant of the Modeste. He was,
when I saw him, so far advanced as to have no hope, and died in a few days.
I attended a post-mortem examination of the body. Inflammation of the mu-
cous coat, from the stomach to the rectum, with its effects from the first stage,
to perfect gangrene existed. The liver was pale, otherwise healthy. It was a
pure case of dysentery.
He, with Lord Joceylyn on board, who filled the office of Military Secretary
to the Admiral, happened to be under my care. Lord J. had a complicated form
of disease?very severe intermittent, but which only existed as a secondary dis-
ease. He had decided congestion about the liver?dysentery intermitting with
a very confined state of the bowels?a great appetite alternately with loathing of
food. I attacked the liver first, deeming that the root of the disease lay there.
Had 30 leeches on with good effect, and slightly touched him with mercury: after
"which, tonics did a great deal. He left the ship pretty well; but when he got any
thing well, he was so very careless, that he had frequent relapses. I recommended
him strongly not to remain in the Tropics, and he has been since invalided.
The Nosological Return shews 36 cases of abdominal disease, and indeed
those cases under the head of Fever had generally also some determination to the
visceral organs. None of them were marked by any peculiar severity, and all
now are doing well.
I had also under my care a Chinese, the master of a junk, who was shot by
pne of our seamen. The master had made a complaint of the seamen for pillag-
ing on board the junk?the seaman belonged to a boat, which boarded and de-
tained the junk. The officer of the boat threatened to complain of the seaman?
the seaman took a loaded musket and fired at the Chinaman, who was distant
from him six yards; the ball took effect on the head, struck him about the site
of the posterior inferior angle of the parietal bone on the right side, penetrated
the bone, passed directly through the brain for the space of about two inches,
and passed out again through the vitreous portion of the temporal bone. I saw
the man half an hour after the injury; he was quite insensible, with dilated pu-
pil; on re-action setting in, I took blood from the arm, during the abstraction of
which, he began to show signs of consciousness. The wound, at either end of
oi
596 Extra-Limites. [Oct. 1
which were portions of brain coming away, I lightly dressed with simple dress-
ing and cold wash, gave smart cathartics, and enjoined perfect quietude. He
was able to be up and walk about in two days, and, in three days, was removed
on board the Volage (we being ordered to sea). We got into a gale of wind, the
deep rolling of the ship hurt him much, and he was unable to rest in a cot?I
tried him, but he rolled out. After this he began to sink, and expired twelve
days after the injury. He was conscious to a couple of days to his death.
I have had another, and almost equally extraordinary injury of the skull, only
with a different result, as he is doing, and likely to do, well. Mr. Olive, the
second Master of the ship, while in a gale of wind lying at anchor, was attend-
ing the compressor in veering cable. In bringing-to to check the cable, the
tackle gave way, the non-compressor recoiled with tremendous force, and in its
sweep, struck Mr. Olive on the left temple. He was thrown, insensible. I saw
him immediately, and found a depression big enough to put one's fist into, deep
as an oyster-shell. There was no external wound; tumefaction coming on, con-
vinced me some of the large temporal branches were ruptured. Without wait-
ing, I instantly took 40 ozs. of blood, which checked further effusion. He lay
insensible five days?is now up and able to walk about?his intellect changed,
but I think not much deteriorated?the tongue, right hand, and arm partially
paralysed. I purpose invaliding him and giving him a certificate for the wound.
I have the honor to be, Sir,
Your obedient humble Servant,
B. VERLING, Surgeon.
Plague in a British Man-of-war, not contagious.
Admiralty, 26th April, 1841.
A CIRCUMSTANCE has occurred regarding the health of two ships in the Medi-
terranean Squadron of so unusual, and I may add, unexpected a nature, as will
I hope justify me in bringing it under the notice of the Board of Admiralty.
It is known to their Lordships that the Zebra was driven on shore in a gale of
wind at Kaiffa on the Coast of Syria on the 2nd of December last, and the creW
having been enabled to land, were lodged in the town, which is described as be-
ing most filthy with all kinds of abominations; it was not however known that
any febrile disease prevailed at Kaiffa, but several cases of plague appeared at
Acre, at a little later period:?some part of the crew were afterwards employed
in assisting to repair the fortifications at St. Jean d'Acre, where, the surgeon
states, " they generally enjoyed good health, with the exception of a few cases
of fever, two of which appear to have died with malignant symptoms;"?'previ-
ous to this, however, there had been ten cases of continued fever in the Zebra,
while on the other parts of the Coast of Syria, all of which recovered, and like
the other ships of the Squadron, nine or ten had been attacked with dysentery*
one of which died.
On the 17th February the Castor arrived at Kaiffa with orders to break up the
Zebra, and embark her crew. The Castor had in like manner previously suffer-
ed from fever and bowel complaints, but not to any extent, and only one death
had taken place from the latter disease.
On the 20th February, fourteen men belonging to the Zebra were sent on
board the Castor in exchange for a party of artificers, and about five days pre-
vious to this, the party employed at St. Jean d'Acre returned to the Zebra.
On the evening of this day, William Thomas, a seaman belonging to the Ze-
bra, who had been employed in the boats at Acre, was attacked with fever, and
1841] Plague in a British Man-of-JVar. 597
died on the afternoon of the 22nd, and between that day and the 25th, thirteen
more cases were added, including an artificer from the Castor. The whole of
these, with the exception of Thomas, who died previously, were immediately
removed on board the Castor, The remainder of the crew were embarked on the
following day, when the Castor sailed for Malta.
The disease with which the men sent from the shore had been attacked, now
manifested unequivocal symptoms of Plague, and every means was used to pre-
vent intercourse with the healthy, eleven men being selected to attend solely up-
on the infected persons.
It is satisfactory to state that not one of these men were attacked with Plague,
nor were any of the Castor's people, except the artificer, who had been landed
and lived with the Zebra's men at Kaiffa, though there were at least twenty-four
persons, including four medical officers, fully exposed to the contagion during its
continuance on board the Castor.
In addition to the usual symptoms of a highly malignant fever, which need
not be recited here, these men had all glandular swellings in the groin or axilla,
and one had carbuncle, which sloughed extensively, and several of the buboes
suppurated.
I beg to add the two first Cases, shewing the progress and symptoms of the
disease.
Kaiffa Bay, 22nd February.
John Barry, first class boy, aged 19, complained this morning of severe head-
ache, burning heat and dryness of skin; eyes watery and dull; countenance de-
pressed; great languor and listlessness, and a disposition to lethargy, with some
confusion of ideas; indistinctness of memory, and hesitation in answering ques-
tions; prostration of strength, and pain in the back; tongue white and dry in
the centre; pulse 100, rather full; says he was taken ill last night.
23rd. Passed an extremely restless night; great prostration of strength; coun-
tenance more lively and less lethargic; there is an enlarged gland in the upper
part of the thigh, the pain of which he complains greatly of; it is painful to the
touch, but there is no external inflammation; pulse 100, irregular and easily
compressed; tongue dry in the centre, with two fervid streaks on each side; to-
wards the apex there is a small shining spot, about the size of a shilling, which
has the appearance a red congested part has, the moment blood has been pressed
from the cutaneous vessels.
p. m. The pain in the groin is his great source of complaint; there is decidedly
less stupor and lethargy, and his ideas are now quite collected; eyes watery, but
expressive; pulse small and thready; occasional vomiting.
24th. Moaned a great deal in the night; complains very much of the bubo,
?Which is slightly enlarged; the skin has become softer and the tongue moist,
and he is perfectly collected and sensible. From this time the pulse gradually
became weaker, and soon after 2 p. m. he was attacked by convulsions, then be-
came comatose, and expired about a quarter before three o'clock.*
Case 2nd.?John Terliven, carpenters' crew of H.M.S. Zebra, aged 29, was
taken ill on the evening of the 22nd February, complaining of headache, slight
Pain of the back and limbs, with great weariness and prostration of strength,
stupor and lethargy, with inclination to sleep, and impatience when aroused,
answering questions in an abrupt manner; in short, there appears an utter in-
difference and apathy to everything around him. Eyes suffused, pupils natural;
skin dry and harsh, but no preternatural heat; thirst; tongue smooth, dry in the
* This lad had returned from St. Jean d'Acre on the 12th February, and had
attended a sick messmate who died that day under suspicious circumstances.
598 Extra-Li mites. [Oct. 1
centre and tremulous, having the peculiar appearance described in the last case;
pulse 100, small and irregular; the symptoms were preceded by shivering.
24th. There is less stupor, and he says he feels better; tongue moist; skin
softer; pulse irregular; a bubo was discovered in his left groin, or rather in the
upper part of his thigh.
25th. Last night the symptoms in general appeared more favorable, and he
expresses himself better, and he has recovered from the state of lethargy. There
is, however, a peculiar and indescribable appearance of anxiety about him;
bowels rather relaxed, but he has the appearance of increased strength.
The pulse became weaker and was not distinguishable at the wrist for some
hours before death; stimulants had not the smallest effect on the pulse, and,
about an hour before he expired, he drew attention to his feet, which were cold
and damp; he said he felt dead as high as his knees, and soon quietly departed,
the bubo remaining stationary.
I beg to annex on the other side, a List of those attacked, with their com-
mencement and discharge.
The preceding Narrative and Cases are taken from the Official Reports of the
Surgeons of the Castor and Zebra, and I beg to add that I have purposely for-
borne to make any comments thereon, being convinced that the facts are of too
important a nature not to command attention.
W. BURNETT,
Inspector-General, &c.
The Secretary of the Admiralty.
1841] Plague in a British Man-of-TVar. 599
a e
s S
<3
NAMES
Age
Quality
Disease
When
taken ill
Of what
Ship
If employed at Aere
for the entire period,
or occasionally in
Boats
Date of
Discharge
into
Castor
Died
Duty
Convales-
cent
1841
Feb. 22
John Barry
John Terlevin...
Edward Epps
Thos. Chaplain..
Wm. Powell
Stwt.Robertson..
John Roberta....
Henry Boyd
Geo. Campbell...
T. Knight....
John Cranfield ..
William Lambert
William Cook
Boy
Capt. Crew
Boy
Ord.
Artificer
Rl. Marine
Qr.-Master
Boy
A. B.
A. B.
Qr.-Master
Boy
Rl. Marine
Pestis
Pcstis
Pestis
Pestis
Pestis
Pestis
Pestis
Pestis
Pestis
Pestis
Pestis
Pestis
Pestis
On Castor
On shore
On shore
On shore
On shore
On shore
On shore
On shore
On shore
On shore
On Castor
On Castor
On Castor
Zebra
Zebra
Zebra
Zebra
Castor
Zebra
Zebra
Zebra
Zebra
Zebra
Zebra
Zebra
Zebra
At Acre all the time
Do. nearly all the time
At Acre in Dec. last
At Acre all the time
Artificer landed from
Castor
Not at Acre
Occasionally in boats
at Acre
At sea all the time
Occasionally in boats
Ditto ditto
All the time at Acre
Ditto ditto
Not at Acre
20th Feb.
22d
S2d
22 d
22d
23d
23d
23d
23d
23d
24th Feb.?at 4 a.m.
25th ? at loh. 30m. a.m.
24th ? at 11 80 p.m.
Duty
27th Feb.?at 3 a.m.
26th Feb.?at 7h. 30m. a.m.
r Convalea-
V, cent
Duty
2d Mar.?at 0 a.m.
26th ? at 7h. 30m. a.m.
8th ? at 6 a.m.
7th ? at 4 15 a.m.
Duty
Note by the Editors.
If anything stronger than this Document can be necessary to prove the idle fear3 of contagionists, it must be a sign in the sky,
like that which Constantine shaped his course by!!
600 Extra-Limited [Oct. 1
NEW HYDRAULIC MACHINE.
We have seen the apparatus to which the following letter refers, and think it is
a most ingenious invention, and well calculated to answer the objects for which
it is intended.?Editors.
Ipswich, September 15th, 1841.
Dear Sir,?In your last, you ask me how your New Hydraulic Machine is
approved of at the different hospitals. I shewed it at Cambridge Hospital to
the house-surgeon and several others of the profession?they all expressed them-
selves astonished with its powers: next I produced it at the hospital at Bury
St. Edmunds, and at the house of Dr. Hake, before Drs. Smith, Probett, Rev.
Mr. Hastie, and several others of the committee?they will order one immedi-
ately you can send them testimonials of its approval by the Humane Society.
At Norwich, Yarmouth, and at this place, all concur in approving of the instru-
ment. Dr. Forbes, of Norwich, says, that not an hospital in England, or a
ship in Her Majesty's service, should be without one.
It will be useless for me to proceed farther at present, as the sanction of the
Royal Humane Society must be obtained before you can get orders.
\ I am, dear Sir,
Your's, &c.
H. PUTLAND.
APPENDIX TO BIBLIOGRAPHICAL RECORD.
ADVICE TO THE DEAF.
39. The present State of Aural Sur-
gery ; being the Substance of a Lecture
delivered at the Royal Dispensary for
Diseases of the Ear. By J. Harbison
Curtis, Esq. Second Edition, 1841.
40. Practical Observations on Inju-
ries of the Head. By William Sharp,
F.R.S., &c. &c. Senior Surgeon to the
Bradford Infirmary. Octavo, pp. 168.
Churchill, London, 1841.
41. The Oily Acids, forming the first
Supplement to the Seventh Edition of
Dr. Turner's Chemistry. By Justus
Liebig, M.D., and W. Gregory*
M.D. Octavo, pp. 121. Taylor and
Walton. Sept. 1841.
42. Statement of Facts, &c.&c. By
Mr. Yearsley, M.R.C.S.
43. The Naturalist's Library. By
Sir W. Jardine, Bart. Ichthyology-
Vol. II. Fishes of Guiana. By Robert
H. Schombuck, Esq. W. H. Lizars,
Edinburgh, 1841.
INTELLIGENCE.
Dr. L. Koecker.
The degree of Doctor of Dental Surgery has been conferred on Dr. L eonard
Koecker, of Conduit Street, by the Baltimore College of Dental Surgery.
IS41 ] On the Poison of Fever. 57
The following are the data upon which the proof of the connexion of diseased
mucous follicles with the peculiar effects of a morbid poison upon the biliary
excretion, may be rested.
1st. It may be considered as admitted, that the special characters of sub-
stances fitted for assimilation are absence of active chemical qualities, and the
capability of yielding to transformations ; and that every substance may be
considered as nutriment, which loses its former properties when acted on by the
vital principle, and does not exercise a chemical action upon the living organ.
2ndly. That in the progress of the functions of nutrition, certain chemical
and organic substances are produced, and from time to time are present in the
blood ; which products it is the office of the different excreting organs to discharge
from that fluid,?the relative activity of these organs depending partly upon the
matter to be eliminated, and partly on other circumstances; thus we have seen
that, in one situation the lungs assume a disproportionate activity, in others, the
liver, &c.
The foregoing propositions being admitted, the following may be regarded as
convertible from them. If substances be introduced into the blood which are
not capable of assimilation or of affording nutriment?whether from their
chemical qualities or from their condition (of decomposition)?it will follow
that, instead of these suffering the transformations which food undergoes to
become assimilated, the blood will undergo their transformation and disease
will be produced.
Also, that numerous modifications in the composition and condition of the
compounds, produced from the elements of the blood, may be the immediate
result of the introduction into it of these substances, and a change in the qua-
lity of the excretions may thus be the first indication of the action of the poison,
as well as of the effort made to expel it.
Numerous facts and observations tend to shew that, in the case of organic or
putrid poisons, the liver is the organ by whose excretions an attempt is made to
rid the blood of the new products thus formed in it.
As first?by a reference to the experiments of injection of putrid pus, &c.,
into the veins of animals, performed by Magendie, Gaspard, Cruveilhier, &c., it
will be seen that when the animal recovered it was after copious discharges of a
vitiated character from the bowels ; to these discharges the last-named writer
attributes the recovery, and adds, that it is a fundamental fact of pathology
that the intestinal canal is chiefly affected in diseases caused by miasmata.
Again, if we refer to the published cases of poisoning from putrid ingesta,
we see that, besides those of irritant poisoning in which the rapid rejection of
the substances was followed by recovery, there is another class in which, after
an interval allowing of the absorption of the poison into the circulation, a diffe-
rent set of symptoms followed, as in the following from Dr. Christison's work
on poisons :?"A family of five persons took for dinner broth made of beef,
which owing to its black colour the master of the family had previously said to
his wife he thought bad and unfit for use.
In the course of some hours two boys were attacked with sickness and vo-
miting, but appear to have got soon well, probably from the early discharge of
the poison. Next morning a washer-woman, who had dined with the family,
Was seized with violent pain in the bowels, diarrhoea, racking pains, and weakness
in the limbs, and did not recover for ten days. On the evening of the second
day the master of the house was similarly affected and was ill for a fortnight.
And a day later, his wife was also seized with a similar disorder, preceded by
soreness of the throat and tongue and difficulty of swallowing, and ending fa-
tally in fourteen days."
It is worthy of notice that the severity of these cases was in proportion to
the interval allowed for absorption of the poison?altogether their resemblance
to the description of the symptoms of typhoid fever, quoted from Louis, is re-
58 Extra-Limitks. [Oct. 1
roarkable. If we enquire why the mucous glands of the lower portion of the
ileum, are more than other parts of the intestine liable to suffer from this pecu-
culiar derangement of the biliary excretion, we shall see reason to think the
cause is the same as would explain their existence in greater number there than
elsewhere, and that this is probably owing to the fact of a second digestion or
chymification being performed in the caecum, during which, it is believed by
some physiologists,* that the entrance to the large intestine is closed, and bile
collected in the lower portion of the small intestine, which does not enter the
caecum till the secondary chymification is completed. The effect of such a re-
tention of an acrid and depraved secretion must be obviously to produce irrita-
tion in the part subjected to its influence, and the same deranged products of
secretion, continuing through much of the duration of the fever, we can account
for this affection not seeming to be limited to any portion of that period, but
why commencing with it,?frequently even preceding it, it ordinarily survives
the continuance of the most prolonged disease.
This explanation also accounts for the disease in these glands being found
farthest advanced nearest to the termination of the intestine?for perforation
occurring almost invariably close to the caecum?for the lymphatic glands of the
mesentery, corresponding to the diseased follicles, becoming diseased?and for
the severity of these affections bearing a direct proportion to the severity of the
fever, and, as it would appear, to the amount of the poison imbibed into the
system.
This view of the relation of diseased follicles to the action of the septic poison
differs both from that which regards dothinenteritis as the cause of fever, and
from that which assigns to it a merely secondary place in this affection. To the
latter are opposed the extremely frequent, and early occurrence of abdominal
symptoms (as diarrhoea) in the typhoid form of fever. With the post mortem
appearances in subjects examined at an early stage ; while the former is irrecon-
cilable with the occasional absence of the lesion, the frequent want of corres-
pondence between its amount and the gravity and fatal result of the fever,
(a correspondence which should exist if the other phenomena of fever were
but the sympathies of the affection of the follicles,) with the occasional per-
sistence of the local disease after the fever has subsided, and with the presence
(almost equally frequent in typhoid fever) of other lesions which cannot be con-
sidered sympathetic of this, but must be ascribed either to the immediate ope-
ration of the poison, or to that state of the blood produced by it; such are
the softening of the spleen, liver and heart, and the inflammatory affection of the
brain and thoracic viscera.
These pathological changes, as well as the derangements of function consti-
tuting the febrile state, will probably be best explained by some such hypothesis
as that advanced by Dr. Hodgkin, which supposes the febrile state to depend
upon a suspension, or at least very considerable interruption of that process by
which, during health, the various parts of the system are continually undergoing
a change, the old materials being removed while others are substituted in their
place."f This hypothesis will be found perfectly in accordance with that of the
* Schultz quoted by Miiller.
f Lectures on Mucous Membranes, Lect. 23.
Dr. Hodgkin's hypothesis seems to explain the great difference in the fatality
of fever as affecting the higher and lower classes of society, since by the mode
of life of the former, more nutriment being taken into the system, and more
organic matters constantly present in the blood, an arrest of the process by
which they are eliminated, must naturally be followed by more complete deterio-
ration of the mass of circulating fluid, and more serious injury to the functions
and structure of the organs supplied by it.
IS4IJ On the Poison of Fever. 59
action of a morbid poison upon the blood, since it will be the natural effect of
the molecular change produced in that fluid by the decomposing particles of the
poison so to modify it as to render it unfit to undergo the capillary attractions
constituting the processes of interstitial absorption, nutrition, and secretion;
and thus instead of Dr. Smith's formula of the order of successive derange-
ments in fever?namely, derangement of innervation?then of circulation, and
lastly of secretions and of the animal fluids ; the more correct one will probably
be, first the molecular change in the blood, then the suspension or modification
of the interstitial processes?or change of particles?then certain derangements
of innervation and of the heart's action, and the result?formed fever.
According to this view, dothinenteritis is one of the phenomena of the second
stage in the action of the poison, and immediately consequent upon certain
modifications of the biliary (and probably also the intestinal) secretion. Its
occurrence cannot be considered essential to typhoid fever, as the contamination
of the blood may cause the molecular changes upon which the foregoing hypo-
thesis supposes lever to depend without this?albeit its absence in typhoid fever
if very rare?while, on the other hand, it may exist without fever necessarily
following, for we frequently see that, of a number of individuals exposed to the
same source of miasm, some will suffer an attack of typhoid fever, while others
will be affected with diarrhoea or dysentery : a fact which is explained by a
reference to those experiments of Gaspard, in which the recovery of the animal
after putrid injection, was attended with profuse and offensive discharges?
seemingly the mode of relieving the blood from the presence of the poison.
Reference has been already made to another set of cases, in which dothinen-
teritis occurs without fever : those namely, in which the poison was so concen-
trated as to produce a rapidly fatal effect, and where examination after a diarrhoea
of only a day or two shewed the same peculiar affection of the follicles as in
typhoid fever; the inference from such cases taken conjointly with those of
fever from the same causes without dothinenteritis, must be that this lesion is
neither cause nor effect of the fever, but a concurrent and contingent effect of the
poison. Most of the other pathological changes of fever are to be explained by
the alterations in the constitution of the blood. Such is evidently the cause of
the softening of the spleen so invariably present in typhoid fever, and such a
little consideration will shew to be the cause of the congestive character of the
typhoid inflammations : for the occurrence most likely to follow such a change
in the molecular attractions of the blood as will interfere with its capillary cir-
culation, is stagnation in this part of the system, of which the consequences are
venous congestion, passive haemorrhages, and the softening of parenchymatous
organs. The stagnant character of the typhoid pneumonia has been remarked
by many; thus Dr. Williams says, " it may be almost a question whether
in these cases the local disease in the lungs is not rather a congestion of
blood in an altered state than an inflammation, and it is very commonly the
sequel rather than the cause of the fever,"*?an opinion which seems fully
warranted by dissection, as well as consistent. Huxham, with the modifications
of the physical signs in this form of disease,+ was so much struck with this
connexion of the local affection and diseased blood, that he compared the state
of the latter in these cases to the scorbutic habit; and Andral countenances this
analogy in the following passages. " The ataxo-adynamic fever recognises for
its commencement some alteration of the blood, whether this alteration may
have taken place spontaneously, and produce a sort of acute scorbutus, or it
may follow the introduction of deleterious agents, as miasms, virus, matters in
a state of putrefaction ; these agents after having modified the composition of
* Article Pneumonia, Cyclop. Prac. Med.
"f See my Observations on Typhoid Pneumonia, Dublin Journal, V. 7> for
several dissections of this disease.
60 Extra-Limiths. [Oct. 1
the blood come to poison the nervous centres. Then the disease is everywhere,
where blood and nerves are to be found, and in every part lesions may occur
which perform but a secondary part in the production of the symptoms.*
Again?" Congestions of the parenchymatous tissues and membranes are
tolerably frequent during the course of fevers. These congestions seem to
depend on the rupture of equilibrium between the globules and fibrine ; they
are very frequent in typhoid and typhus fevers, and small-pox ; the spleen and
other parenchymatous tissues are usually congested in these diseases, and a
diminution of fibrine as compared with the globules (whether absolute or
relative) is the alteration of the blood observed in these maladies.
" The ancients concludedtfrom the phenomena just mentioned that in the
diseases in question, the blood is altered, and that its elements have a great
tendency to separate. They designated by the phrase putridity, that morbid
condition in which the vital powers seem to yield to physical causes, and the
blood becomes putrescent. Borden, whose opinions as to the nature of typhoid
fever are remarkably sagacious and philosophical, does not hesitate to consider
that malady as connected with a general condition of the system, which he
designates by the name of acute scurvy. This phrase is not inaccurate, so far
as regards the condition of the blood. A diminution of the quantity of the
coagulable material of the blood is a general fact observed in all great febrile
disturbances; thus in miasmatic fevers there is first absorption of the miasma,
and immediately after, the only prominent phenomenon is an alteration of the
blood. This alteration which occurs in typhoid fever is the effect of some cause
as yet unknown.
Haemorrhage is well known to be characteristic of typhoid fever. That from
the air-passages is enumerated by Chomel among the distinctive symptoms of
the disease. Intestinal haemorrhage is also a frequent and an unfavourable
symptom, and .indicative of a diseased state of the blood. This hemorrhage is
preceded by stagnation. The softening of viscera is always observed in con-
junction with an altered and fluid state of the blood. In some descriptions of
softened spleen by Andral and others, the blood contained in it is compared to
the lees of wine.
Sect. V.? Characters of the Disease produced by the Putrid Miasm.
It has been remarked, that a general resemblance may be traced between the
disease produced by the infectious animal poison of typhus and the exanthemata;
its first and most striking analogy being the almost constant presence of a pecu-
liar eruption. Another particular in which it resembles them and differs from
the disease now under consideration is the absence of any constant internal le-
sion ; the pathology of typhus being of a functional or physiological kind, while
that of endemic or typhoid fever is anatomical and precise in its nature. The
distinctive characters of the two affections may be thus stated. In typhus, a
poison is generated by certain changes in the fluids of the living body, which,
being received into the blood of a healthy individual, has a tendency to excite in
that fluid the transformations from which it has itself arisen, and by which it
?will be reproduced : a process during which certain phenomena occur, as that of
* Clinique Medicale Translated, p. 610. Several cases are given, in which
all the symptoms of typhoid fever were produced apparently by mental and
bodily depression, but after death no lesion was discovered?for a striking case
of this acute scorbutus, see Dr. Law's paper before quoted at p. 19.
?f* Lectures on the Blood, reported in Dublin Medical Press, Aug. 11, and
Provincial Medical and Surgical Journal, Aug. 21.
1S4J] On the Poison of Fever. 61
eruption (an effort apparently to free the system from the presence of the poison),
and the conclusion of which is marked by the presence in the excretions of the
material necessary for the generation anew of the disease in any person into
whose blood it may be received. All these phenomena may occur without ap-
preciable change in the structure of any organ, and in fact death may be pro-
duced without any morbid appearance beyond that degree of congestion natu-
rally connected with the modification of its processes of nutrition and secretion.
In typhoid fever the events following the introduction of the putrefactive
poison are different; it will appear upon examination into these, that no new
material of reproduction is generated, that the eruption is not a true exanthema
or identical with that of typhus, being later in its appearance, less constant,
more scanty, consisting of successive crops rather than persistent and uniform,
as in typhus. A marked modification of the molecular changes of the system
occurs in this as in every variety of febrile movement, but its continuance is
evidently less uniform than in typhus, being subject to alternations and remis-
sions, at times approaching those of the periodic fevers ; and the critical change
attending its resolution is more gradual and liable to be less certain and com-
plete, as well as to recur by relapse, unlike that of typhus. But the most im-
portant distinction consists in the fact that the typhoid miasm has, like other
putrid poisons, a tendency to be eliminated from the system through the biliary
excretion, in the course of which process a peculiar form of irritation is set up
in the alimentary canal, while no such tendency can be asserted of the poison of
true typhus, in the majority of cases of which the biliary excretion suffers rather
a diminution than otherwise.
But it may be said it is by no means proved that the typhoid affection of Louis
and Chomel is of endemial origin, and in order to establish the connexion be-
tween miasm as a cause, and fever characterised by dothinenteritis as the effect,
either this must be proved, or it must be made to appear that the fevers which
in our own country may be traced to this source are to be distinguished from
typhus by the intestinal lesion.
With regard to the French typhoid fever, we are led to infer this conclusion
from the following facts : the existence of such miasm in abundance in the fosses,
wells, and river of Paris?the almost invariable occurrence of gastro-intestinal
affections in those newly-arrived there?the fact that typhoid fever attacks the
same class so constantly as to make a change of circumstances regarded as one
of the essential causes of the disease?and lastly, the testimony of the most
distinguished physicians that it is not propagated by contagion. Let the expe-
rience of our large hospitals, with reference to the infection of typhus, be com-
pared with the following statement of Andral. " In Paris, either in the hospi-
tals or out of them, we never recognized in this disease (dothinenteritis) the
slightest appearance of a contagious character. In the hospitals we do not see
it transmitted from the individual who brings it from without to those who are
lying in the beds next his own ; neither do we see that the patients who lie in a
bed previously occupied by a person who has recovered from, or who has died of
a dothinenteritis, are attacked by it'; neither are the physicians or medical students
"who come there attacked with it, more particularly those who have had to
come in contact with patients labouring under the disease. Out of the hospitals
what circumstances are more favourable to contagion than those generally found
combined in the case of medical students who attend their companions when
affected with typhoid fever ? Shut up in a room which in general is very small,
they pay them the most assiduous and devoted attention night and day ; if the
affection were contagious almost all of them would contract it, and yet we do
pot remember to have seen the disease even once arise in this way in a healthy
individual."*
* Spillan's Clinique Medicale, p. 728.
62 Extra-Li mites. [Oct. 1
Louis does not mention contagion in his observation on the causes of the
disease, but Dr. Gerrhard states that, in conversation, he informed him that he
had never seen a case so communicated.
But the question may be elucidated by an examination of some of the pub-
lished histories of fevers occurring in this country from exposure to endemial
sources, from which it will be seen that in numerous instances these were found
to be attended with the characteristic dothinenteritis of the French typhoid affec-
tion. Thus in London, after making every due allowance to the advocate of the
exclusive infectiousness of typhus, we must contend that the writings of Dr.
Armstrong, Dr. Southwood Smith, and others, prove the frequent occurrence
of continued fever from these causes, while the treatise of Dr. Smith shews how
large a proportion of the fever of London is of the intestinal kind?having all
the characters, symptomatic and anatomical, of the " typhoid affection." A
similar remark may be made of Dr. Tweedie's work, which contains numerous
cases of dothinenteritis, while it affords strong indirect testimony to the endemial
source in the statement as to the period of the year at which the disease pre-
vailed, and its remarkable subsidence under the influence of low temperature,
rain, and frost?causes which exert a precisely opposite effect upon contagious
typhus.*
* Dr. Tweedie, who is by no means a strong advocate for the malarial origin
of fever, remarks, that " cold and wet Summers are always remarked to be com-
paratively healthy, while disorders of the bowels in such seasons are seldom ob-
served. The number of patients admitted into the Fever Hospital, in the
Autumn months of the last three years, establish this principle. In August,
September, and October, 1827, there were 205; in the same months of 1828,
the numbers were 170; in the Autumn of 1829, only 94 were received. The
cause of this progressive diminution is undoubtedly to be traced to the cold wet
Summers of the last two seasons."
A similar remark has been made by many physicians as to Dublin ; thus Dr.
Percival says, " it has long been observed, that protracted dry weather is pecu-
liarly productive of fever in Dublin ; and that rainy weather agrees best with
the general health of its inhabitants." And while he states that the worst forms
of typhous fever prevailed at an advanced period of the Winter, and were cha-
racterised by cerebral congestion, he thus distinctly characterizes the endemial
fever : " But the seat of peculiar congestion in the autumnal fever was the inner
surface of the intestines, and sometimes the mesenteric organs. The type of
this epidemic was more irregular than any other; its invasion more obscure ; its
progress and duration less defined. The subjects of the disease were often
broken down and declining constitutions, in which the digestive organs had been
long impaired, &c." Could any description more resemble that of the dothin-
enteritis of Louis ? Dr. Davidson, who argues for the identity of the two
fevers, meets the above statement of Dr. Tweedie thus :?" An opinion exactly
opposed to that of Dr. Tweedie is given by Dr. Armstrong. He states, that in
England, typhus is evidently favoured by a low temperature, being most prevalent
in the cold seasons of Winter and Spring, generally abating or disappearing, as
the heat of Summer advances, and often prevailing to a considerable degree in
cold wet Autumns." This passage is extracted from his work on Typhus, in
which Dr. Armstrong advocated the doctrine of contagion. At a later period of
his life he taught the exclusively malarial origin of fever, and in his lectures ad-
duces in proof of that doctrine the great prevalence of fever in London during
hot seasons, and particularly during the dry hot Summer of 1818. The only
mode of reconciling these opposite opinions of the same observer, is by supposing
that he described the nature of each epidemic correctly as it was presented to
him, but not being prepared to recognize a distinction in their nature, was natu-
1841] On the Poison of Fever. 63
Again, while Dr. Addison ascribes the numerous cases of synochus and typhus
presented at Guy's Hospital to river malaria, Dr. Bright and Dr. Hodgkin
prove the identity of many of the cases received into that institution with the
disease described by Louis. Similar observations have been made in other
places. In Birmingham, dothinenteritis is stated to be the constant mofbid ap-
pearance of the few cases of fever which occur; and on referring to Dr. Ward's
account, already quoted (p. 43), of a fever which he clearly shews to have arisen
from river malaria, we find it stated that it was present in all the fatal cases.
In Dublin, Dr. Cheyne marked three periods occurring in his experience,
during which the contagious typhus usually prevalent, gave way to epidemics of
intestinal fever, in which evidence of malaria was frequently met with, but infec-
tion not so?the pathology was that of dothinenteritis. These observations are
confirmed by others; thus, Drs. Graves and Stokes have published a number of
cases of peritonitis from perforating ulcer of the ileum, occurring during one of
these periods, 1826-29. Dr. Kennedy states that the glands of Peyer were found
by him to be more or less diseased, in a large proportion of the cases of the
same period, presenting, as he remarks, a striking contrast in this respect to the
fever (contagious typhus) of 1837.* Dr. Stokes also says, "In the epidemic of
1826 and 1827, we observed the follicular ulcerations (dothinenteritis of the
French) in the greater number of cases. In many instances perforation took
place, and the whole group of vital and cadaveric phenomena corresponded al-
most exactly to the dothinenteric affection of the French authors."f
We meet with similar evidence of two fevers in Glasgow. In 1836, says Dr.
Stewart, I was much struck with the simultaneous occurrence in the wards of
the Glasgow Fever Hospital, of two sets of cases in which the symptoms (how-
ever little most of them might seem to differ when viewed individually) presented,
when taken collectively, characters so marked as to defy misconception, and to
enable the observer to form with the utmost precision the diagnosis of the nature
of the disease and the lesions to be revealed by dissection. More particularly
it was remarkable to observe, that while in the one disease the affection in those
who presented no eruption was so slight and of so short duration as to make it
very questionable whether it deserved the name of typhus, and while the fatal
cases presented an abundant and generally a profuse eruption ; those labouring
under the other, which equally and even in a much higher proportion/ went on
to a fatal termination, rarely presented any, and then only a very scanty eruption.
It was further remarkable, that while in the one several successive patients had
either been restored to health or fallen victims to the severity of the affection,
the disease under which those laboured who lay side by side with them, though
characterized by much less urgent symptoms, pursued its gradual course through
weeks and months consecutively, and in the majority of cases to a fatal issue.
And finally it was more remarkable still, that to complete the contrast already so
striking, dissection proved the existence in the one disease of most extensive
local lesions, in the other, the absence of all prominent local lesion whatsoever.
Dr. Stewart adds, "that during the Summer and Autumn of 1836, the cases of
typhoid fever were numerous, but from the month of November in that year, (at
which time both the type and amount of typhus became more formidable) till
June, 1838, not more than a dozen cases, if there were even so many, and these
at long intervals, were admitted for treatment."J
This evidence is pretty clear as to the existence of two forms of disease. As
rally led by the evidently non-contagious character of the last observed, to doubt
the correctness of his views of the origin of the first.
* Medical Report of the Cork Street Fever Hospital.
f Lectures, Lond. Med. and Surg. Journal.
J On Typhus and Typhoid Fevers, Edin. Med. and Surg, Journ. No. 145.
64 Extra-Limitks. [Oct. 1
to the causes, the highest authority in Glaagow on this subject, Dr. Cowan,
writing during the period referred to, and with the cases before him, says?" many
of the cases of the production and propagation of disease must be ascribed to
the habits of our population, to the total want of cleanliness among the lower
order of the community, to the absence of ventilation in the more dense'tv peopled
districts, and to the accumulation for weeks and months together of filth of every
description in our public and private dunghills, to the over-crowded state of the
lodging-houses resorted to by the lowest classes, and many other circumstances
unnecessary to mention."
In Edinburgh, according to Dr. Christison, " the intestinal affection has re-
peatedly presented itself in groups?the constitutio dothinenterica, to speak in
nosographical language, has repeatedly appeared and disappeared as a subordi-
nate or intercurrent epidemic, in the course of the more general epidemic?
typhus." And according to Dr. Reid, such cases occur not unfrequently in
the country parts of Scotland, and are occasionally sent to the Edinburgh In-
firmary.
In Liverpool we are informed they occur in an intercurrent way, as in Glas-
gow,* and we need only refer to the evidence before the committee on the health
of towns for proof of sufficient endemial causes.
In the Navan Fever Hospital there have been for the last seven years almost
always two distinct forms of fever present, one or other occasionally preponde-
rating, so as at times nearly to exclude the other. Thus for the first three years
the prominent features were pain, tenderness, and meteorism of the abdomen,
diarrhoea, and not unfrequently these symptoms combined with catarrh ; several
cases of perforation of the ileum occurred towards the close of this period ;
petechise were not frequent and were late in their appearance, and we ha'l
few instances of communication by contagion. During the three following
years a highly contagious fever prevailed, and the symptoms and treatment were
completely different, delirium, subsultus, dysphagia, being the ordinary symp-
toms, and diarrhoea being rarely met with ;?nearly every case presented the
measly efflorescence, and instances of contagion were as numerous as they had
been rare previously. During the present Summer the prevailing type has been
the abdominal fever of the first period, and instances of typhus are infrequent,
certainly not a fourth of the whole, and sent exclusively from a district in which
the epidemic of last year still lingers.
In America the existence of two kinds of fever has been maintained by Dr.
Jackson and Dr. Gerrhard. The former says, in his report on typhoid fever, " it
is plain that there are at least two species of continued fever, both in Europe
and in this country, and further researches may very possibly shew more."
Dr. Gerrhard states, that "from the information we possess we should con-
jecture that the two diseases (British or Irish typhus and dothinenteritis), are
widely different in their symptoms, anatomical characters, treatment, and mode
of transmission."
The following extracts from' his able paper will shew that the two forms of
fever exist in America at different periods and with distinct characters, just as in
our own cities.
"Dothinenteritis is by no means a rare disease in Philadelphia, although less
common than at Pari3. In the essay alluded to, I established the identity of the
anatomical characters and of the symptoms of the fever occurring at Philadelphia
with that observed at Paris.. .. The typhous fever which is so common throughout
theBritish dominions, especially in Ireland, is not attended with ulceration or other
lesion of the glands of Peyer For a period of at least ten years, there has
been no epidemic of this nature at Philadelphia. In the year 1827, a large
* See Dr. Lombard's Letter, Dub. Med. Journal, Vol. X.
1841J On the Poison of Fever. 65
number of Irish emigrants were ill of a typhoid fever with ulceration of the
small intestines, which was probably dothinenteritis, and during several suc-
cessive years there were more or less extensive epidemics of remittent and
intermittent fevers occurring in the neighbourhood of the city, but not often
extending into the central parts of the town, in the Winter of 1835-6, a form
of fever not commonly met with at the hospitals was observed from time to
time. It was characterized by pungent burning heat of the skin, dusky aspect
of the countenance, subsultus, delirium, with great stupor and prostration, but
there was no diarrhoea, and but few symptoms referrible to the alimentary canal.
It was the disease which afterwards appeared as an epidemic The
evidence of contagion was direct and conclusive; three of the principal
nurses and about a dozen assistant nurses, besides a number of patients ill with
various diseases were taken with the fever. There was only one nurse of a
ward in which many of the patients were collected who escaped ; but several
of his assistants and patients were taken ill. The wards in which the fever-
patients were placed were large and well ventilated. The contrast between the
two fevers in this respect (their infectious character) is obvious Season
of the Year.?The epidemic began in March and continued until August?
there were a few scattering cases afterwards. The Summer was unusually
cool, and the Spring and Winter cold Pathological Anatomy.?In
this large number of autopsies, amounting to about fifty, there was but in
one case, and that doubtful in its diagnosis, the slightest deviation from
the natural appearance of the glands of Peyer The fact that
the morbid changes pathognomonic of dothinenteritis are not met with in the
typhous fever, would of itself seem conclusive that the two diseases are no more
identical than pneumonia and pleurisy. Although in some respects the two
affections are analogous and even similar; the radical difference of anatomical
lesions is at least as well marked as the distinction between the symptoms. It
is indeed singular that there should be of late a strong tendency to confound
two fevers which were regarded as entirely distinct by some of the older phy-
sicians."
In the above quotations we see strongly marked the differences of the two
affections as to prevailing season?symptoms, pathology, and mode of trans-
mission, and the similarity of each to one or other of the two forms of European
fever.
Having endeavoured to collect and arrange the testimony of the best autho-
rities as to the sources of the fever poison, we stop upon the threshold of the
extensive inquiry into the laws which regulate the diffusion of the disease in an
epidemic form.
To attempt this would require the fullest investigation into the differences and
analogies of the two affections, their modes of combination in the same indi-
vidual, and their occurrence in an intercurrent mode during the same epidemic
period, all of which modifications of disease would be found reconcileable with
the theory of two poisons; the one having its elements in the blood, and repro-
duced in it; the other a product of putrefactive decomposition, and not
reproduced in the human body; while on the other hand Dr. Davidson's recent
essay contains in itself proof that his own theory of a single typhoid poison is
not tenable, since it involves the assertion of the identity of two diseases, one
of which (according to him) requires to be kept up by an uninterrupted
series of cases of contagion, while the other, according to the best observers,
never propagates itself by contagion at all. In short, according to this doctrine,
we must believe that the same poison, shall at the same time and place, and
among the same collection of individuals, produce two diseases totally dissimilar
in their mode of access, symptoms, pathology, treatment, and mode of trans-
mission.

				

